# Asthma incidence in children growing up close to traffic: a registry-based birth cohort

**DOI:** 10.1186/1476-069X-12-91

**Published:** 2013-10-26

**Authors:** Anna Lindgren, Emilie Stroh, Jonas Björk, Kristina Jakobsson

**Affiliations:** 1 Division of Occupational and Environmental Medicine, Lund University, Lund, Sweden

**Keywords:** Air pollution, Asthma, Bronchitis, Children, Environmental, Epidemiology, GIS, Nitrogen oxides, Roadway proximity, Traffic

## Abstract

**Background:**

Recent reviews conclude an association between traffic-related pollution and incidence of asthma in children, but not all studies agree. Studies have almost exclusively relied on parental-reported symptoms or parental-reported diagnoses of asthma and wheeze. Our aim was to investigate if traffic exposure is associated with higher incidence of early onset asthma, using registry-based outcome data.

**Methods:**

We investigated a birth cohort in southern Sweden, consisting of N = 26 128 children with outcome and exposure data (born July 2005–2010). Of these children, N = 7898 had additional covariate information. The cohort was followed to the end of 2011.

Traffic intensity, and dispersion-modeled concentrations of NO_X_ (100×100 m grid), at residential addresses, were linked with registry data on dispensed asthma medication (the Swedish Prescribed Drug Register), and hospital and primary health care diagnoses of bronchiolitis, obstructive bronchitis and asthma (The Scania Health Care Register).

Covariate information was obtained from questionnaires distributed to parents at Child Health Care-centre visits, eight months after birth. Cox proportional hazards regression was used for the statistical analyses.

**Results:**

Living in close proximity to a road with ≥8640 cars/day (compared to 0–8640 cars/day), was not associated with higher incidence of first purchase of inhaled β_2_-agonist (adjusted hazard ratio (adj.HR) = 0.9, 95% CI: 0.8-1.0); third year purchase of inhaled β_2_-agonist (adj.HR = 0.7, 95% CI: 0.6-0.9); bronchiolitis (adj.HR = 0.7, 95% CI: 0.6-0.9), obstructive bronchitis (adj.HR = 1.0, 95% CI: 0.9-1.2), or asthma (adj.HR = 0.7, 95% CI: 0.6- 0.9). Similar results were found for inhaled corticosteroids, and in relation to NO_X_.

**Conclusions:**

Traffic-related exposure was not associated with higher incidence of asthma medication, or diagnoses of asthma, bronchiolitis, or obstructive bronchitis, in children 0–6 years in southern Sweden. This may depend on the low levels of traffic pollution in the area, mainly well below the WHO-guideline for NO_2_.

## Background

It is well known that traffic-related air pollution can trigger asthma symptoms in children and adults [1]. There is also increasing evidence that long-term exposure to traffic exhaust increases the incidence of asthma development in children. Recent reviews conclude that living close to a major road is associated with higher asthma incidence in children, although there is not evidence to conclude a casual relation [2,3].

Asthmatic symptoms in children, sometimes termed “wheeze” or “obstructive respiratory symptoms”, has diverse etiology. Before the age of 3 years, asthmatic symptoms are mainly due to respiratory virus infections, while after 3 years, asthma due to allergic sensitization is more often the cause [4]. Early asthmatic symptoms, “wheeze”, to some degree predict later asthma [5]. Traffic has been connected to both early [6,7], and late childhood asthma incidence [8-12].

For children, asthmatic symptoms, “wheeze”, are not clinically distinct disease entities, but rather clinically similar wheezing symptoms which becomes classified according to age and other characteristics. Bronchiolitis is a diagnosis of wheeze mainly used for infants. Obstructive bronchitis is a diagnosis used for single episodes of wheezing symptoms for children mainly younger than 3 years, when nothing speaks for allergic etiology. Asthma is a diagnosis often used for a third episode of wheezing symptoms, or for a first episode when the child is older, the parents are known to be allergic, or the child has had atopic eczema which speaks for an allergic heredity [13]. The above statement refers to common diagnostic practice in Sweden but practice may differ between countries.

The first line of treatment for obstructive wheezing symptoms is inhaled β_2_-agonists, which is prescribed for all of the mentioned diagnoses and give immediate relief by dilating the airways. Inhaled corticosteroids, which has a more preventive anti-inflammatory effect, is prescribed as an additional medication for treatment of repeated wheeze or wheeze with suspected allergic component and is considered more specific for asthma. Some studies have used inhaled β_2_-agonists/corticosteroids as a proxy for asthma incidence [14,15].

Studies on long-term effects of traffic on asthma, however, have traditionally relied on parental-reports of wheeze or parental-reports on diagnosis of asthma. Clinical asthma examinations cannot be performed in small children due to compliance difficulties [16], and have not been performed in cohort studies on traffic pollution and asthma incidence in older children either, probably because of the cost and effort required which would result in a small sample size. Due to the risk of awareness bias in parental-reported data [17], and the risk of overestimation of effects in small samples [18], there is a need for registry-based studies which can have both objective outcome data and a larger sample size.

The Swedish Prescribed Drug Register has a complete (99.7%) coverage of individual-level dispensed medication for all individuals living in Sweden, and dispensed asthma medication will in this study be used as a proxy variable for incidence of asthma. We also used diagnosis of bronchiolitis, obstructive bronchitis, and asthma, from the Scania Health Care Register (SHCR), which covers inpatient and outpatient care in the region, from hospitals as well as primary health care centers. However, the SHCR has less complete coverage and will therefore be used as a secondary outcome. This is the first study to use dispensed medication to estimate long-term effects from traffic-related exposure on asthma, and only one study has used hospital and primary health care registries for this purpose before [8].

The overall study aim was to investigate if children growing up close to high traffic intensity or high levels of nitrogen oxides (NO_X_) are at higher risk of developing asthma or other obstructive respiratory disease, “wheezing”, in early childhood.

## Methods

### Study area

Scania is the southernmost county of Sweden, with a population of 1 243 329 in year 2010 [19]. Children born in Scania, whose mothers were registered as living in the municipalities Malmö, Svedala, Vellinge or Trelleborg were included, since survey data with covariate information were available from Child Health Care centers (CHC) in this area. Malmö is the major municipality in the county, 298 963 inhabitants, with a large socio-economically disadvantaged immigrant population, 30.2% foreign born. Previous studies have found that immigrants,and children residing in areas with low income, has a higher exposure to NO_2_ in Malmö [20,21].

Malmö has the highest level of air pollution in the area. Although pollutant levels in the region are low in a European context (Additional file 1), they are higher than in most of Sweden, due to long-range transport of pollutants from the continent and extensive harbor and ferry traffic.

### Selection of study population

**Figure 1:**
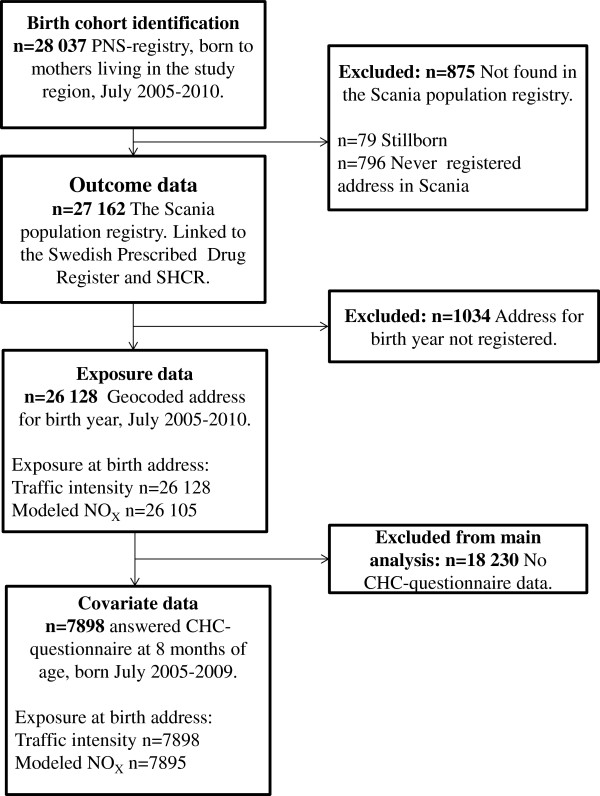
Selection of study population.

A flow-chart of the study population selection is displayed in Figure 1. The study was limited in time to children born from July 2005, since individual level medication data is only available since then. All children were followed to the end of 2011.

To identify a birth cohort, we retrospectively retrieved the identity number of all children born by mothers living in Malmö, Svedala, Vellinge and Trelleborg during July 2005–2010, from the Perinatal Revision South (PNS)-registry. The PNS registry has a 100% coverage of visits on obstetric and peri/prenatal units in the county. Out of 28 037 children identified in the PNS-register, 875 were not found in the Scania population registry and thus excluded, since they were not registered as living in the region during childhood. Outcome data was available for all children found in the Scania population registry (n = 27 162), by linkage to the SHCR and the Swedish Prescribed Drug Register. Geographical coordinates (geocodes) for registered birth year address was available for 26 128 children, for which exposure was assessed. Most of the missing geocodes belonged to children born in December, whose late birth date probably lead to addresses not being registered during year of birth. Geocodes were retrieved for birth year and subsequent years for each child, until the end of 2010. Finally, covariate information from questionnaires routinely distributed at Child Health Care centers was available for 7898 children, which formed the main study cohort.

### Ethical permission

This study was approved by the Lund University Ethical Committee (registration no. 2011/468). No formal informed consent was required, but the study was advertised in the local newspaper and information was distributed to Child Health Care centers, allowing parents to request that their children not be included in it. No such request was raised.

### Asthma medication

The Swedish Prescribed Drug Register includes all drugs dispensed at pharmacies in Sweden, since July 2005 linked to personal identity numbers [22]. The registry is maintained by the National Board of Health and Welfare. All expedited drugs on the pharmacies are registered, with a very small number of incorrect or incomplete registrations of ID. The population coverage with correct patient identities is 99.7% [23].

The registry contains data on all dispensed prescriptions in ambulatory care. Over-the-counter (OTC) medications and drugs used at inpatient settings are not included. Medication data are classified according to the Anatomical Therapeutic Chemical (ATC) Classification System [24].

The Pharmaceutical Benefit Scheme, which is mainly tax financed, covers the main costs for drugs in ambulatory care in Sweden. There is a ceiling on the total amount that a patient pays during a 12-month period for subsidized pharmaceuticals (2013: SEK 2200, €252). The drug costs of children younger than 18 years, living in the same household, are counted together.

We obtained information on medications prescribed for obstructive airways disease (ATC-code R03). The outcomes used were dispensed prescription of inhaled β_2_-agonist (ATC-codes: R03AC, R03AK04, R03AK06, R03AK07), and inhaled corticosteroids (ATC-codes: R03BA, R03AK06, R03AK07). Drugs with code R03AK06 and R03AK07 are combinations of β_2_-agonists and corticosteroids and therefore occur in both outcomes.

As primary outcomes we used:

1) Incidence of first ever dispensed inhaled β_2_-agonist.

2) Incidence of third year with dispensed inhaled β_2_-agonist.

3) Incidence of first ever dispensed inhaled corticosteroid.

4) Incidence of third year with dispensed inhaled corticosteroid.

First dispensed medication was seen as a proxy for incidence of obstructive respiratory disease, but may reflect primarily transient disease. Third year with dispensed medication was seen as a proxy for more persistent disease. The three years were not necessarily consecutive years.

### Diagnoses of bronchiolitis, obstructive bronchitis, and asthma

In Sweden, all healthcare consultations are recorded in county-specific databases. The SHCR holds details for primary health care, and hospital based in- and outpatient care for Scania. In Sweden, all patients are registered to a general primary care practice. However, patients are not obliged to attend primary care before seeing a specialist, although that is the most common procedure [25].

Each consultation generates data entries that are transferred to SHCR and which constitute the basis for reimbursement to the healthcare providers. The hospital care has a good coverage and validity for diagnostic codes [25,26]. However, for primary care, the number of consultations with diagnostic codes is less complete. The diagnostic codes from public care are transferred to SHCR, but have some missing registration of diagnostic codes due to incomplete journal entries. For private health care providers, consultation events, but not diagnostic codes, are transferred to SHCR. Private care makes up approximately 30% of all primary care in Scania [25].

The number of visits that lacked diagnostic codes was only provided on overall level for the children in this study, not individual level.

The hospital-based health care uses a Swedish version of the diagnostic ICD-10 system, ICD-10-SE, and the primary health care uses a simplified version, KSH97-P. As secondary outcomes we used diagnostic codes from SHCR, including hospital-based as well as primary health care. Visits are often given multiple diagnostic codes, but we included only the primary diagnostic code.

The secondary outcomes were primary diagnoses of:

1) Bronchiolitis (J210, J218, J219),

2) Obstructive bronchitis (J200-J209, J22-P)

3) Asthma (J450- J459, J45-P, J469)

### Exposure assessment

Geocodes for the children’s officially registered residential addresses were retrieved from the population registry, for each year from birth until the end of 2010. Individuals are positioned at the center coordinate of their residence.

### Traffic intensity

A Geographical Information System based registry, from the Swedish National Road Database, provided data on traffic intensity on all major roads in the county. To assess exposure to traffic, we identified the road with the heaviest traffic intensity within 100 m of the residence. Traffic intensity was categorized as “no road”, “road with 0–2880 cars/day”, “2880–8640 cars/day”, “8640–14400 cars/day”, and “≥14400 cars/day”, based upon daily (24-hour) mean levels.

The traffic intensity categories were merged into a dichotomous variable, “0-8640 cars/day” (including children with “no road”) and “≥8640 cars/day”, to obtain enough power, since not enough cases lived in the highest exposure category to assess it separately. The classification was based upon results from previous studies in the same geographical region, which found a higher prevalence of asthma among adults living within 100 m of roads with ≥8640 cars/day [27,28]. Separate analyses were done in relation to traffic intensity for: 1) birth address exposure 2) birth address exposure, with children censored when/if they moved during time at risk.

### Modeled concentrations of NO_x_

Concentrations of NO_X_ (NO_2_ + NO) at each child’s residential address, were modeled as annual means for each calendar year 2005–2010, with a spatial resolution of 100×100 m. Concentrations were obtained from an emission database (EDB) for NO_X_, previously described in detail [20]. The emission sources included were: road traffic, shipping, aviation, railroads, industries and larger energy and heat producers, small-scale heating, working machineries, working vehicles and working tools. Background levels of NO_X_ due to transport of pollutants from the continent, were also included, based on data from rural background monitor stations, and meteorological factors were incorporated. For dispersion calculations, the EDB was combined with a modified Gaussian two-dimensional dispersion model (AERMOD). Bilinear interpolation was applied. Validation of the EDB showed satisfying agreement between modeled and measured concentrations of NO_2_ (Spearman’s r = 0.8) [29].

Separate NO_X_ -analyses were done for: 1) birth address exposure 2) birth address exposure, with children censored when/if they moved during time at risk, and 3) mean NO_X_ during all years at risk (excluding 2011 for which geocodes were not available). The mean NO_X_ during time at risk was only assessed for those never moving outside the study area during time at risk. Since time at risk differ with outcome, the number with modeled mean NO_X_ during time at risk, also vary depending on outcome.

We used a categorical classification of NO_X,_ since previous studies in the same geographical region have indicated non-linear relationship between NO_X_ and asthma [27,28]. We based our categories on exposure contrasts (≤15, 15–25 and >25 μg/m^3^), rather than on the distribution of NO_X_ among the population.

### Covariate information

The final cohort for the main analysis included 7898 children born in the region, whose parents had answered a CHC center questionnaire 8 months after birth. The questionnaire was handed out to parents in Malmö, Svedala, Vellinge and Trelleborg, in conjunction with their children’s 8 month checkup at the CHC centers [30]. The questionnaire had been validated and translated from Swedish into five different languages: Albanian, Arabic, English, Serbo-Croatian, and Somali. The response rate varied between years but was approximately 65% of handed out questionnaires [30].

Variables considered for inclusion in the multivariable models were: sex, birth weight, smoking during pregnancy, environmental tobacco smoke (ETS), mold at home, parental allergy, furred pets at home, breastfeeding, parental origin, parental education, problems to pay bills, type of housing, and birth year.

### Statistical analysis

All statistical analyses were performed using SAS, version 9.3. Survival analysis was performed because of different lengths of follow-up of the children. We used two different censoring variables: 1) children were censored at year of study end (2011), or 2) children were censored when they moved from their original birth address, or at year of study end (2011).

Descriptive Kaplan-Meier survival curves, with numbers at risk, were displayed for all outcomes. The proportional hazard assumptions for exposure and outcome were checked graphically by log(−log(survival))-curves. We then reported unadjusted Cox proportional hazards-ratios (Cox PH) between exposure and outcomes.

We used prescreening of variables in combination with a stepwise Cox PH-procedure, to select covariates to include in the final multivariable models. We performed the same selection procedure for all outcomes in relation to traffic intensity, to find the most important predictors. Any variable staying in any of the outcome models, was included in all the models, for model consistency. Traffic intensity was forced to remain in the model in each step. The following steps were done:

1. Univariable prescreening of all covariates in Table 1, except city. Any variable with a univariate p-value < 0.2 for the HR between the covariate and the outcome, was selected to next step.

**Table 1:** Description of the main cohort, n = 7898

		**N (%)**	**HR (95% CI)**^**a**^
			**1st purchase, inhaled β**_**2**_**-agonist**
Sex	Girl	3784 (49)	1.0
	Boy	3996 (51)	1.3 (1.2–1.5)
	Missing	118	
Birth weight	2500–4000 (normal)	6079 (78)	1.0
	500–2499 (low)	301 (4)	1.1 (0.9–1.5)
	4001–6500 (high)	1396 (18)	1.1 (1.0–1.3)
	Missing	122	
Smoking during pregnancy	No	7275 (94)	1.0
	Yes	499 (6)	1.2 (1.0–1.5)
	Missing	124	
Environmental tobacco smoke	No	6591 (85)	1.0
	Yes	1177 (15)	1.2 (1.1–1.4)
	Missing	130	
Breastfeeding	≥8 months	3920 (56)	1.0
	<8 months	2807 (40)	1.2 (1.1–1.3)
	Never breastfed	278 (4)	1.3 (1.0–1.6)
	Missing	893	
Parental allergy	No	3177 (46)	1.0
	Yes	3751 (54)	1.2 (1.1–1.4)
	Missing	970	
Furred pets at home	No	5790 (75)	1.0
	Yes	1922 (25)	1.0 (0.9–1.1)
	Missing	186	
Mold at home	No	7326 (95)	1.0
	Yes	386 (5)	1.0 (0.8–1.3)
	Missing	186	
Problems to pay bills	Never or seldom	7361 (96)	1.0
	Yes, >6 months/year	348 (5)	0.8 (0.6–1.0)
	Missing	189	
Swedish parents	Yes, both Swedish	4811 (62)	1.0
	One foreign	1290 (17)	0.9 (0.8–1.1)
	Both foreign	1689 (22)	0.8 (0.7–1.0)
	Missing	108	
Highest education any parent	>12 years	5612 (73)	1.0
	9–12 years	1792 (23)	1.1 (1.0–1.3)
	≤ 9 years	297 (4)	1.1 (0.8–1.4)
	Missing	197	
Type of housing	Owned house	2783 (36)	1.0
	Tenant-owned apartment	2242 (29)	0.9 (0.8–1.0)
	Rented apartment	2616 (34)	0.9 (0.8–1.1)
	Other	101 (1)	0.7 (0.4–1.1)
	Missing	156	
City	Vellinge	449 (6)	1.0
	Svedala	664 (8)	0.9 (0.7–1.2)
	Trelleborg	611 (8)	1.1 (0.8–1.4)
	Malmö	6134 (78)	0.8 (0.6–1.0)
	Missing	40	
Birth year	2005	1066 (14)	1.0
	2006	2395 (30)	1.1 (0.9–1.3)
	2007	1664 (21)	1.0 (0.8–1.2)
	2008	2179 (28)	1.0 (0.9–1.2)
	2009	594 (8)	1.2 (0.9–1.5)

^a^Unadjusted.

2. All the selected variables were included into a multivariable Cox model, together with traffic intensity which was forced to stay in the model. Backward selection was performed, with significance level for staying (Slstay) = 0.1.

3. Starting with an initial model including the variables selected from step 2. Forward selection was performed, with significance level for entry (Slentry) = 0.2, to consider for inclusion the variables initially not selected at step 1.

4. Starting with the model selected from step 3. Finetuning was done by stepwise selection- Slentry/Slstay 0.05. The variables selected to be included in the final multivariable models were: Sex, ETS, breastfeeding, parental allergy, parental origin, parental education and year of birth.

Since all selected covariates approximately fulfilled the PH-assumption, we used the Cox PH model for the final multivariable analyses, to assess the incidence of asthma medication and diagnoses in relation to traffic-related exposures. Multivariable analyses presented do not include children with missing values for any of the variables included.

We also performed sensitivity analyses: we analyzed the unadjusted relation between traffic-related exposure and outcomes, for all children with complete information on exposure and outcome (n = 26 128). For the main cohort (n = 7898), we separately estimated effects for Malmö vs. the remaining study area, to see if results were consistent across geographical regions. We also performed an analysis excluding children born 2006, the year when most children had high traffic exposure. Finally, we performed analyses restricted to children with high socio-economic status (n = 3464), here defined as children whose parents fulfilled all the following criteria; never problems to pay bills, at least one parent with >12 years education, and both parents born in Sweden. The question about ability to pay bills was here not dichotomized as in the main analysis, but instead a finer original classification was used, where “never problems to pay bills” was separated from “seldom problems to pay bills”.

The hazard ratios (HR) in all analyses were displayed with 95% confidence intervals (CI).

## Results

### Covariate description

Population characteristics, and incidence of inhaled β_2_-agonists in relation to these characteristics, are displayed in Table 1. Most of the risk factors included in the multivariable analyses, were more common in proximity to roads with low traffic intensity (parental allergy, ETS, no or little breastfeeding, short parental education). Male sex of the child was associated with high traffic intensity. Parental origin, and year of birth had no consistent relation to traffic, but a large proportion of the children with high traffic intensity and high NO_X_ were born in 2006.

### Exposure description

**Figure 2:**
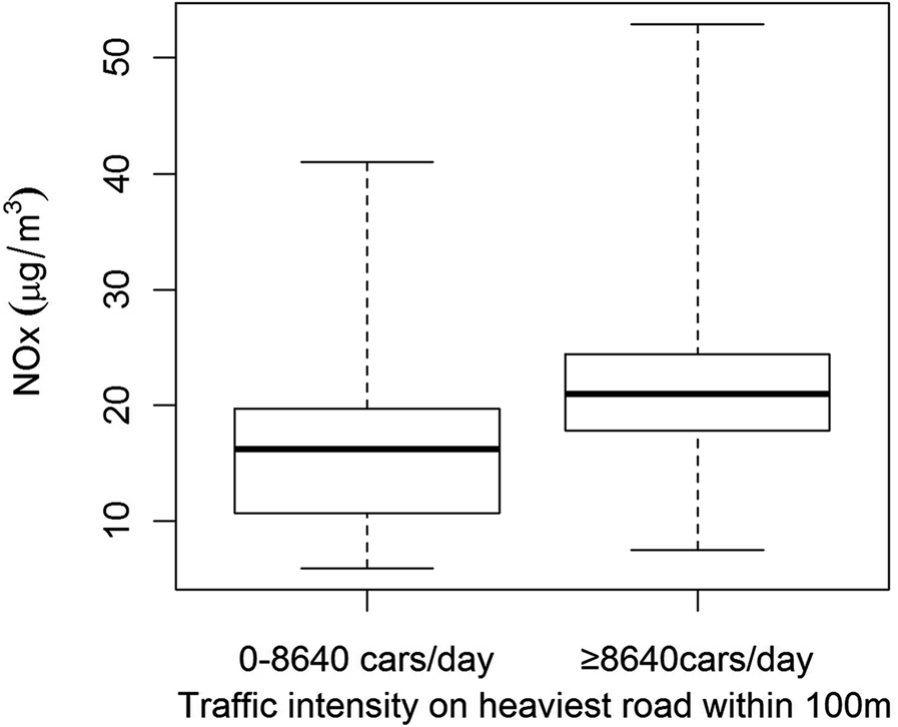
**Distribution of modeled annual mean NO**_**x **_**at birth address, by traffic intensity (n = 7895).** Upper and lower borders of boxplots represent the 75th and 25th percentiles and the bold line is the median. The whiskers extend to the minimum and maximum of the NO_x_-concentrations.

The percentage of the study population living ≤100 m from a traffic intensity of 0–8640 cars/day at birth address was 73.8%, compared to 26.2% with traffic intensity of ≥ 8640 cars/day at birth address. We classified modeled NO_X_ levels into ≤15, 15–25 and >25 μg/m^3^. For exposure at birth address, the percentage of the population living in respective category was 34%, 57% and 9%. Mean NO_X_ at birth year was 17 μg/m^3^, and the percentile distribution was 9.2, 11.8, 17.6, 21.1, and 24.6 μg/m^3^ (10th, 25th, 50th, 75th, and 90th percentile). Min, Max = (6.1, 45.9) μg/m^3^. The distribution of NO_X_ by traffic intensity, is displayed in Figure 2.

### Missing outcome data and Kaplan-Meier survival curves

Diagnostic codes were available for 97% of the hospital visits, and for 50% of the primary care visits. Among the latter, 70% of public primary care visits had diagnostic codes, while codes were completely missing for private primary care visits. The proportion of private primary care was 28% of total primary care visits.

Kaplan Meier survival curves, and life table data, showed that most of the incidence of first dispensed asthma medication and diagnoses, occurred in age 1–2 years (Additional file 1: Table S5 and Additional file 2). The oldest children were followed to age 6 years.

### Incidence of dispensed medication

Incidence of purchased inhaled β_2_-agonist, and inhaled corticosteroids, was lower for children living close to a road with ≥8640 cars/day (compared to 0–8640 cars/day) at birth address (Table 2). Both first and third year purchase was associated with a lower traffic intensity, in some cases significantly so. Similar results were observed in relation to NO_X_. The results were consistent for children who never moved during time at risk, for mean NO_X_ during time at risk, and both before and after adjustment for covariates (Table 2, and Additional file 1).

**Table 2:** Adjusted HR (95% CI) for asthma medication and diagnoses, in relation to traffic-related exposure, n = 7898

	**Inhaled asthma medication**^**a**^				**Diagnoses**^**a**^		
	**β**_**2**_**-agonist**	**β**_**2**_**-agonist**	**Corticosteroid**	**Corticosteroid**	**Bronchiolitis**	**Obstructive bronchitis**	**Asthma**
	**1st purchase**	**3rd year**	**1st purchase**	**3rd year**			
**Heaviest road ≤100 m, birth address**^**b**^							
0–8640 cars/day	1.0	1.0	1.0	1.0	1.0	1.0	1.0
≥8640 cars/day	0.9 (0.8–1.0)	0.7 (0.6– 0.9)	0.8 (0.7–0.9)	0.8 (0.6–1.0)	0.7 (0.6–0.9)	1.0 (0.9–1.2)	0.7 (0.6–0.9)
**Heaviest road ≤100 m, never moved**^**b**^							
0–8640 cars/day	1.0	1.0	1.0	1.0	1.0	1.0	1.0
≥8640 cars/day	0.9 (0.7–1.0)	0.7 (0.5–1.0)	0.8 (0.6–0.9)	0.9 (0.6–1.2)	0.7 (0.6–0.9)	1.0 (0.8–1.2)	0.7 (0.6–0.9)
**NO**_**X, **_**birth address**^**c**^							
≤15 μg/m^3^	1.0	1.0	1.0	1.0	1.0	1.0	1.0
15–25	0.8 (0.7–1.0)	0.7 (0.5–0.8)	0.8 (0.7–0.9)	0.7 (0.5–0.9)	0.6 (0.5–0.8)	1.1 (0.9–1.3)	0.8 (0.7–0.9)
>25	0.7 (0.6–0.9)	0.6 (0.4–0.8)	0.7 (0.5–0.9)	0.6 (0.4–0.8)	0.5 (0.4–0.8)	1.1 (0.8–1.4)	0.7 (0.5–0.9)
**NO**_**X**_**, never moved**^**c**^							
≤15 μg/m^3^	1.0	1.0	1.0	1.0	1.0	1.0	1.0
15–25	0.8 (0.7–1.0)	0.6 (0.4–0.8)	0.8 (0.7–0.9)	0.6 (0.4–0.8)	0.6 (0.5–0.8)	1.1 (0.9–1.3)	0.8 (0.7–0.9)
>25	0.8 (0.6–1.0)	0.5 (0.3–0.9)	0.6 (0.5–0.8)	0.5 (0.3–0.9)	0.5 (0.4–0.8)	1.1 (0.8–1.5)	0.7 (0.5–0.9)
**NO**_**X, **_**lifetime mean**^**d**^							
≤15 μg/m^3^	1.0	1.0	1.0	1.0	1.0	1.0	1.0
15–25	0.8 (0.7–0.9)	0.7 (0.6–0.9)	0.7 (0.6–0.9)	0.7 (0.6–0.9)	0.7 (0.5–0.8)	0.9 (0.8–1.1)	0.7 (0.6–0.9)
>25	0.8 (0.6–1.1)	0.5 (0.3–0.9)	0.6 (0.5–0.9)	0.5 (0.3–0.9)	0.7 (0.5–1.0)	1.0 (0.7–1.3)	0.7 (0.5–1.0)

^a^adjusted for sex, ETS, breastfeeding, parental allergy, parental origin, parental education, and year of birth.

^b^n = 6007 children had complete covariate information and traffic exposure assessments.

^c^n = 6005 children had complete covariate information and modeled NO_X_ concentrations.

^d^number of children with complete covariate information and modeled mean NO_X_ during time at risk varies between n = 5194-5248 depending on outcome.

### Incidence of bronchiolitis, obstructive bronchitis and asthma

There was a significantly lower incidence of diagnoses of bronchiolitis, and asthma, but not obstructive bronchitis, among children living close to a road with ≥8640 cars/day (compared to 0–8640 cars/day) at birth address (Table 2). Similar results were observed in relation to NO_X_. The results were consistent for children who never moved during time at risk, and for mean NO_X_ during time at risk. The results were consistent before and after adjustment for covariates, except the HR for obstructive bronchitis, which diminished with adjustment (Table 2, and Additional file 1).

### Sensitivity analyses

An analysis of all children for which outcome and exposure data was available (n = 26 128), unadjusted for any factors, showed that traffic-related exposure was statistically significantly associated with a lower incidence of all outcomes except obstructive bronchitis, for which the HR was not significantly different from 1 (Additional file 1).

For the main cohort (children with covariate information, n = 7898), we stratified our analyses separately for Malmö vs. the remaining region*,* and the results were largely consistent across the regions (data not shown). The results were also consistent when excluding children born 2006. Finally, we performed an analysis restricted to children with high socio-economic status (n = 3464) and the results for this subgroup were similar to the results for the main cohort (Additional file 1).

## Discussion

There was no increased purchase of asthma medication or diagnosis of bronchiolitis, obstructive bronchitis or asthma among children 0–6 years, growing up close to a road with high traffic intensity, or high levels of NO_X_. On the contrary, there was a lower incidence for all outcomes except obstructive bronchitis, among these children. This indicates that traffic-related exposure is not a risk factor for early onset asthma/wheeze in children in southern Sweden.

### Strengths and limitations

A strength of the study was the register-based outcome data with complete coverage of dispensed medication, which prevents potential awareness bias due to parental-reported outcomes. There are still some possibilities of selection bias due to questionnaire data in this study, since the confounder information was only available through CHC-questionnaires, which is likely to have lead to a selection towards high socio-economic status among those who answered the CHC-questionnaire. A potential limitation was that the drug register only includes dispensed medication. The observed lower incidence of medication among children in households with bad economy or with immigrant parents in the present study, raise a suspicion they cannot afford to dispense prescribed medication (or do not get diagnosed in the first place), to the same degree as the children in households with good economy. A previous study in Swedish children found low socio-economic status to be related to higher incidence of late onset wheeze, when based on self-reported data of diagnosis or wheeze [31]. However, a recent Swedish study did not find income to be a predictor for dispense of drugs, after controlling for health status, but there was higher prescription rate toward people with high education [32]. However, since we had individual level data on socio-economic status we could address this by adjustment and restriction on different socio-economic indices, which did not affect the result, and thus this is not a likely source of bias for the results in our study. Another limitation was that a non-negligible percentage of the health care visit data lacked diagnostic codes, which could possibly cause a bias which we cannot account for. This is also why we treated it as a secondary outcome only, which supports the results from the medication data, but cannot be fully trusted on its own.

That the results for the larger study population were the same as for the cohort with questionnaire information strengthens that there is a higher incidence of wheeze in areas with low traffic pollution in the region. It is implausible that air pollution would be “protecting” against asthma, and the results therefore speaks for the presence of unmeasured risk factors, or different health-seeking behavior, in areas with low traffic pollution.

Another strength was the exposure data in this study. Residential addresses for each year since birth were known, which exclude a migration bias which could otherwise be expected to dilute the effects. We also had validated high quality exposure data for NO_x_, modeled with a high resolution, which further minimize the risk of other exposure misclassification biases which could be expected to dilute the effects.

The levels of modeled NO_X_ at children’s home address in this study were low compared to other studies, despite the high resolution of the grid, which can be expected to increase the exposure range [33]. Since different studies use different measures of traffic exposure, complicating comparisons, we also provide a table with background levels of air pollution in the region, to give a picture of the exposure situation in the area (Additional file 1).

We believe the quality of exposure data is better than in most other studies which have found a relation between traffic and wheeze, we therefore see it as unlikely that poor quality of exposure data would be the cause of the negative findings in our study.

### Comparison with other studies

Recent reviews conclude that there is, overall, evidence for a relation between long-term exposure to traffic and asthma incidence in children [2,3]. However, not all individual studies agree, and little differentiation has been made in reviews according to age of asthma onset. We think there is more evidence for an association with persistent wheeze and later onset asthma, than with early onset asthma/wheeze. At least in the studies in the Nordic countries which is where the exposure situation is similar to our study. A cohort study in Norway had similar finding to our study, with a negative association between NO_2_ and early onset asthma (RR = 0.8, 95% CI: 0.6-1.0) [34]. However, late onset asthma (≥4 years age), had a positive but non-significant association with NO_2_. A Swedish cohort study also found no association between NO_X_ and transient early wheeze before 2 years age (OR = 0.8, 95% CI: 0.5-1.4), but a positive association with persistent wheeze [35], and a positive association with asthma onset at age 12 [10].

Some studies outside the Nordic countries have found associations between traffic-related exposures and incidence of wheeze or early asthma [6,7], but others have not [36,37]. In a Dutch birth cohort, NO_2_ was not associated with incident asthma at age 2, but was associated with asthma incidence in older age [9].

In contrast, cohort studies in older children seems to have found more consistent results for traffic-related exposure to be associated with asthma incidence [9-12]. Clark et al. 2010 used hospital and primary care diagnosis records, and found a relation between NO_2_ and asthma incidence already at ages 3–4 years [8]. However, this study had more restrictive case definition of asthma compared to our study, reflecting more severe or persistent asthma.

It should be noted that numerous studies with positive associations between traffic-related exposure and asthma incidence are still statistically non-significant [3]. These studies have in reviews still been interpreted as contributing evidence for a relation between traffic-related exposure and asthma. Our study on the contrary, was based on large numbers and can thus rule out positive effects with high statistical certainty. However, confounding can never be fully excluded. We also want to point out that the exposure levels in our study were lower than most other studies, something which could also explain the lack of effect.

We think that our study together with the results from previous studies in the Nordic countries, suggests that traffic exposure, at the levels observed, either is not a risk factor for incidence of early onset wheeze or asthma, or that the effects may be so small that they are easily overridden by other risk factors. However, the situation may be different in countries with higher exposure to traffic pollution. Also, it should be kept in mind that the results from our study does not exclude effects on late-onset asthma in children, or wheeze that persists into older age. This cohort should be followed up in later age to investigate the relation between traffic and later-onset of childhood asthma, or persistent wheeze continuing into older age.

## Conclusions

We found no association between growing up close to traffic and higher incidence of dispensed asthma medication, diagnosis of bronchiolitis, obstructive bronchitis or asthma, in children 0–6 years in southern Sweden. This indicates that traffic-related exposure is not a risk factor for early onset asthma, or “wheeze”, in southern Sweden, something which may depend on the low levels of traffic-related air pollution in the area.

## Abbreviations

Adj.HR: Adjusted hazard ratio; ATC: Anatomical therapeutic chemical classification system; CHC-centers: Child health care centers; CI: Confidence interval; Cox PH: Cox proportional hazards; EDB: Emission database; HR: Hazard ratio; ETS: Environmental tobacco smoke; NOX: Nitrogen oxides; PNS-registry: Perinatal Revision South-registry; SHCR: Scania health care register; Slentry: Significance level for entry; Slstay: Significance level for staying.

## Competing interests

The authors declare that they have no competing interests.

## Authors’ contributions

AL drafted the manuscript, conducted the statistical analyses, and did the main design of the study and interpretation of results. ES conducted exposure modeling, and participated in the design of the study and interpretation of results. JB participated in interpretation of results. KJ participated in the design of the study and in interpretation of results. All authors made revisions on drafts, and read and approved of the final manuscript.

## Supplementary Material

Additional file 1: Table S1 Urban background. Descriptive data of regional air pollution at monitoring station in Malmö. **Table S2.** Unadjusted HR (95% CI) for asthma medication and diagnoses, in relation to traffic-related exposure, n = 7898. **Table S3.** Sensitivity analysis including all children with outcome and exposure data, n = 26128. **Table S4.** Sensitivity analysis of children with high socio-economic status ^a^, n = 3464. **Table S5.** Life table data. Click here for file

Additional file 2 Kaplan-Meier survival curves of asthma medication and diagnoses in relation to traffic intensity, with number at risk. Click here for file
