# Supplementary material for: Asthma incidence in children growing up close to traffic: a registry-based birth cohort
Source: Environ Health. 2013 Oct 26;12:91. doi: 10.1186/1476-069X-12-91 (PMC4016196; doi:10.1186/1476-069X-12-91)
Supplement: Additional file 1: Table S1 — Urban background. Descriptive data of regional air pollution at monitoring station in Malmö. Table S2. Unadjusted HR (95% CI) for asthma medication and diagnoses, in relation to traffic-related exposure, n = 7898. Table S3. Sensitivity analysis including all children with outcome and exposure data, n = 26128. Table S4. Sensitivity analysis of children with high socio-economic status a, n = 3464. Table S5. Life table data. [file 1476-069X-12-91-S1.docx]

**Additional file 1.**

**Table S1.** Urban background. Descriptive data of regional air pollution at monitoring station in Malmö. Annual mean concentrations measured at Rådhuset Malmö- monitoring station 8773. Data source: IVL Swedish Environmental Research Institute Ltd. Available: http://www.ivl.se/miljo/

| Year | SO_2_ (μg/m^3^) | NO_2_ (μg/m^3^) | O_3_ (μg/m^3^) | PM_10_ (μg/m^3^) | PM_2.5_ (μg/m^3^) |
| --- | --- | --- | --- | --- | --- |
| 2005 | 3.7 | 20.2 | 48.9 | 17.5 | 11.1 |
| 2006 | ^a^ | ^a^ | ^a^ | 18.2 | 12.3 |
| 2007 | 1.4 | 16.7 | 49.7 | 16.6 | 11.4 |
| 2008 | 1.6 | 17.1 | 53.9 | 15.6 | 11.2 |
| 2009 | 1.8 | 16.7 | 49.7 | 14.9 | 12.2 |
| 2010 | 2.1 | 19 | 51.7 | 15.9 | 13.5 |
| 2011 | 1.8 | 17.9 | 57 | 20.8 | 15.8 |

^a^ Measurements were not performed during the whole year, why calender year means were not presented.

**Table S2.** Unadjusted HR (95% CI) for asthma medication and diagnoses, in relation to traffic-related exposure, n=7898.

|  | Inhaled asthma medication^a^ | | | | Diagnoses^a^ | | |
| --- | --- | --- | --- | --- | --- | --- | --- |
|  | β_2_-agonist  1st purchase | β_2_-agonist  3rd year | Corticosteroid  1st purchase | Corticosteroid  3rd year | Bronchiolitis | Obstructive bronchitis | Asthma |
| Heaviest road ≤100m- birth address^b^ | | | | | | | |
| 0-8640 cars/day  ≥8640 cars/day | 1.0  0.90 (0.80-1.02) | 1.0  0.78 (0.62-0.97) | 1.0  0.85 (0.75- 0.97) | 1.0  0.84 (0.67- 1.05) | 1.0  0.75 (0.62- 0.90) | 1.0  1.11 (0.96-1.28) | 1.0  0.80 (0.68-0.93) |
| Heaviest road ≤100m- never moved^b^ | | | | | | | |
| 0-8640 cars/day  ≥8640 cars/day | 1.0  0.90 (0.79-1.02) | 1.0  0.78 (0.58-1.03) | 1.0  0.82 (0.71-0.95) | 1.0  0.87 (0.66-1.15) | 1.0  0.75 (0.62-0.91) | 1.0  1.10 (0.95-1.28) | 1.0  0.79 (0.66-0.93) |
| NO_X-_ birth address ^c^ | | | | | | | |
| ≤15 μg/m^3^  15-25  >25 | 1.0  0.82 (0.74-0.92)  0.73 (0.59-0.89) | 1.0  0.66 (0.54-0.81)  0.62 (0.44-0.87) | 1.0  0.78 (0.70-0.88)  0.70 (0.56-0.87) | 1.0  0.67 (0.55-0.82)  0.63 (0.44-0.88) | 1.0  0.68 (0.58-0.80)  0.61 (0.45-0.84) | 1.0  1.14 (0.99-1.31)  1.25 (1.0-1.57) | 1.0  0.82 (0.71-0.94)  0.71 (0.54-0.92) |
| NO_X_- never moved ^c^ | | | | | | | |
| ≤15 μg/m^3^  15-25  >25 | 1.0  0.83 (0.74-0.94)  0.75 (0.60-0.95) | 1.0  0.58 (0.46-0.74)  0.56 (0.34-0.94) | 1.0  0.79 (0.69-0.89)  0.66 (0.51- 0.85) | 1.0  0.57 (0.44- 0.72)  0.52 (0.31-0.89) | 1.0  0.68 (0.58-0.81)  0.61 (0.44-0.85) | 1.0  1.17 (1.01- 1.36)  1.22 (0.95-1.57) | 1.0  0.82 (0.71-0.95)  0.71 (0.53-0.95) |
| NO_X-_ lifetime mean ^d^ | | | | | | | |
| ≤15 μg/m^3^  15-25  >25 | 1.0  0.75 (0.67-0.84)  0.75 (0.59- 0.94) | 1.0  0.66 (0.54- 0.80)  0.51 (0.31- 0.85) | 1.0  0.71 (0.63-0.81)  0.61 (0.46- 0.80) | 1.0  0.64 (0.52- 0.78)  0.47 (0.28- 0.80) | 1.0  0.66 (0.56-0.78)  0.64 (0.45-0.91) | 1.0  1.02 (0.89-1.17)  0.96 (0.73-1.27) | 1.0  0.75 (0.65-0.86)  0.64 (0.47-0.87) |

^a^ Unadjusted. ^b^ n= 7898 ^c^ n= 7895 ^d^ n=6851-6919 depending on outcome.

**Table S3.** Sensitivity analysis including all children with outcome and exposure data, n=26128.

HR (95% CI) for asthma medication and diagnoses, in relation to traffic-related exposures.

|  | Inhaled asthma medication^a^ | | | | Diagnoses^a^ | | |
| --- | --- | --- | --- | --- | --- | --- | --- |
|  | β_2_-agonist  1st purchase | β_2_-agonist  3rd year | Corticosteroid  1st purchase | Corticosteroid  3rd year | Bronchiolitis | Obstructive bronchitis | Asthma |
| Heaviest road ≤100m- birth address ^b^ | | | | | | | |
| 0-8640 cars/day  ≥8640 cars/day | 1.0  0.90 (0.84- 0.96) | 1.0  0.78 (0.67- 0.91) | 1.0  0.87 (0.80- 0.94) | 1.0  0.84 (0.72- 0.97) | 1.0  0.85 (0.76- 0.94) | 1.0  1.05 (0.96- 1.14) | 1.0  0.84 (0.77- 0.93) |
| Heaviest road ≤100m- never moved ^b^ | | | | | | | |
| 0-8640 cars/day  ≥8640 cars/day | 1.0  0.89 (0.83- 0.96) | 1.0  0.77 (0.63- 0.94) | 1.0  0.85 (0.78- 0.93) | 1.0  0.82 (0.67- 1.0) | 1.0  0.84 (0.75- 0.94) | 1.0  1.04 (0.95- 1.14) | 1.0  0.81 (0.73- 0.90) |
| NO_X-_ birth address ^c^ | | | | | | | |
| ≤15 μg/m^3^  15-25  >25 | 1.0  0.83 (0.78- 0.89)  0.82 (0.74- 0.91) | 1.0  0.68 (0.59- 0.78)  0.68 (0.54- 0.85) | 1.0  0.78 (0.72- 0.84)  0.70 (0.62- 0.79) | 1.0  0.66 (0.57- 0.76)  0.64 (0.51- 0.80) | 1.0  0.75 (0.68- 0.83)  0.58 (0.49- 0.68) | 1.0  1.07 (0.98- 1.17)  0.91 (0.79- 1.04) | 1.0  0.79 (0.72- 0.86)  0.64 (0.56- 0.74) |
| NO_X_- never moved ^c^ | | | | | | | |
| ≤15 μg/m^3^  15-25  >25 | 1.0  0.83 (0.77- 0.89)  0.84 (0.75- 0.93) | 1.0  0.63 (0.53- 0.74)  0.65 (0.48- 0.90) | 1.0  0.78 (0.72- 0.84)  0.69 (0.61- 0.79) | 1.0  0.58 (0.49- 0.68)  0.55 (0.39- 0.78) | 1.0  0.75 (0.68- 0.83)  0.57 (0.49- 0.67) | 1.0  1.08 (0.99- 1.19)  0.87 (0.75- 1.00) | 1.0  0.78 (0.71- 0.85)  0.60 (0.51- 0.70) |
| NO_X-_ lifetime mean ^d^ | | | | | | | |
| ≤15 μg/m^3^  15-25  >25 | 1.0  0.78 (0.73- 0.84)  0.77 (0.68- 0.87) | 1.0  0.66 (0.57- 0.76)  0.63 (0.48- 0.83) | 1.0  0.72 (0.67- 0.78)  0.63 (0.55- 0.73) | 1.0  0.61 (0.53- 0.71)  0.58 (0.44- 0.78) | 1.0  0.73 (0.66- 0.81)  0.57 (0.47- 0.69) | 1.0  1.02 (0.93- 1.12)  0.86 (0.74- 1.00) | 1.0  0.75 (0.68- 0.82)  0.63 (0.54- 0.75) |

^a^ Unadjusted. ^b^ n= 26 128 ^c^ n= 26 105 ^d^ n=18422-18561 depending on outcome.

**Table S4.** Sensitivity analysis of children with high socio-economic status^a^, n=3464. HR (95% CI) for asthma medication and diagnoses, in relation to traffic-related exposure.

|  | Inhaled asthma medication^b^ | | | | Diagnoses^b^ | | |
| --- | --- | --- | --- | --- | --- | --- | --- |
|  | β_2_-agonist  1st purchase | β_2_-agonist  3rd year | Corticosteroid  1st purchase | Corticosteroid  3rd year | Bronchiolitis | Obstructive bronchitis | Asthma |
| Heaviest road ≤100m- birth address ^c^ | | | | | | | |
| 0-8640 cars/day  ≥8640 cars/day | 1.0  0.91 (0.74- 1.10) | 1.0  0.66 (0.45- 0.95) | 1.0  0.77 (0.62- 0.96) | 1.0  0.73 (0.52- 1.03) | 1.0  0.73 (0.53- 1.00) | 1.0  0.98 (0.76- 1.26) | 1.0  0.63 (0.48- 0.83) |
| Heaviest road ≤100m- never moved ^c^ | | | | | | | |
| 0-8640 cars/day  ≥8640 cars/day | 1.0  0.85 (0.68- 1.07) | 1.0  0.52 (0.31- 0.89) | 1.0  0.66 (0.51- 0.86) | 1.0  0.57 (0.34- 0.94) | 1.0  0.73 (0.53- 1.01) | 1.0  1.02 (0.78- 1.33) | 1.0  0.64 (0.48- 0.86) |
| NO_X-_ birth address ^d^ | | | | | | | |
| ≤15 μg/m^3^  15-25  >25 | 1.0  0.88 (0.74- 1.06)  0.78 (0.57- 1.08) | 1.0  0.76 (0.55- 1.05)  0.61 (0.35- 1.04) | 1.0  0.86 (0.70- 1.04)  0.68 (0.48- 0.97) | 1.0  0.72 (0.53- 0.98)  0.63 (0.38- 1.04) | 1.0  0.61 (0.46- 0.80)  0.54 (0.32- 0.89) | 1.0  1.05 (0.83- 1.33)  1.07 (0.72- 1.58) | 1.0  0.81 (0.65- 1.03)  0.59 (0.38- 0.92) |
| NO_X_- never moved^d^ | | | | | | | |
| ≤15 μg/m^3^  15-25  >25 | 1.0  0.91 (0.75- 1.11)  0.84 (0.59- 1.21) | 1.0  0.69 (0.46- 1.02)  0.40 (0.14- 1.10) | 1.0  0.87 (0.70- 1.08)  0.58 (0.37- 0.90) | 1.0  0.65 (0.44- 0.96)  0.39 (0.14- 1.08) | 1.0  0.61 (0.46- 0.81)  0.56 (0.33- 0.94) | 1.0  1.14 (0.89- 1.46)  1.23 (0.80- 1.88) | 1.0  0.80 (0.63- 1.03)  0.68 (0.43- 1.09) |
| NO_X-_ lifetime mean ^e^ | | | | | | | |
| ≤15 μg/m^3^  15-25  >25 | 1.0  0.80 (0.66- 0.96)  0.95 (0.65- 1.41) | 1.0  0.79 (0.57- 1.10)  0.45 (0.17- 1.24) | 1.0  0.75 (0.61- 0.92)  0.70 (0.44- 1.13) | 1.0  0.68 (0.50- 0.94)  0.59 (0.26- 1.35) | 1.0  0.61 (0.46- 0.80)  0.77 (0.44- 1.38) | 1.0  0.88 (0.69- 1.13)  1.20 (0.76- 1.89) | 1.0  0.75 (0.59- 0.95)  0.77 (0.45- 1.32) |

^a^defined as fulfilling the following criterias: swedish-born parents- education of at least one parent >12 years, parents never have problem to pay the bills.

^b^Adjusted for sex, ETS, breastfeeding, parental allergy and year of birth ^c^ n= 2912 ^d^ n= 2911 ^e^ n=2462-2489 depending on outcome.

**Table S5.** Life table data. Incidence of asthma medication, diagnoses, and migration, for different ages, for children who may have moved during time at risk.

| Inhaled B1-agonist, 1^st^ dispense | | | | | | | | |
| --- | --- | --- | --- | --- | --- | --- | --- | --- |
| Age | N at beginning of interval | N Censored | N events | N at risk ^a)^ | Proportion Terminating | CumulativeSurvival | Incidence  (Prob. Density) | Hazard rate |
| 0 | 7898 | 0 | 131 | 7898 | 0.02 | 0.98 | 0.017 | 0.02 |
| 1 | 7767 | 0 | 564 | 7767 | 0.07 | 0.91 | 0.071 | 0.08 |
| 2 | 7203 | 497 | 448 | 6954.5 | 0.06 | 0.85 | 0.059 | 0.07 |
| 3 | 6258 | 1818 | 164 | 5349 | 0.03 | 0.83 | 0.026 | 0.03 |
| 4 | 4276 | 1379 | 72 | 3586.5 | 0.02 | 0.81 | 0.017 | 0.02 |
| 5 | 2825 | 1921 | 38 | 1864.5 | 0.02 | 0.79 | 0.017 | 0.02 |
| 6 | 866 | 857 | 9 | 437.5 | 0.02 | 0.78 | 0.000 | 0.00 |
| Inhaled corticosteroid , 1^st^ dispense | | | | | | | | |
| Age | N at beginning of interval | N Censored | N events | N at risk ^a)^ | Proportion Terminating | CumulativeSurvival | Incidence  (Prob. Density) | Hazard rate |
| 0 | 7898 | 0 | 102 | 7898 | 0.01 | 0.99 | 0.013 | 0.01 |
| 1 | 7796 | 0 | 470 | 7796 | 0.06 | 0.93 | 0.06 | 0.06 |
| 2 | 7326 | 515 | 403 | 7068.5 | 0.06 | 0.87 | 0.053 | 0.06 |
| 3 | 6408 | 1891 | 148 | 5462.5 | 0.03 | 0.85 | 0.024 | 0.03 |
| 4 | 4369 | 1420 | 61 | 3659 | 0.02 | 0.84 | 0.014 | 0.02 |
| 5 | 2888 | 1965 | 28 | 1905.5 | 0.01 | 0.82 | 0.012 | 0.01 |
| 6 | 895 | 892 | 3 | 449 | 0.01 | 0.82 | 0.000 | 0.00 |
| Bronchiolitis, 1^st^ diagnosis | | | | | | | | |
| Age | N at beginning of interval | N Censored | N events | N at risk ^a)^ | Proportion Terminating | CumulativeSurvival | Incidence  (Prob. Density) | Hazard rate |
| 0 | 7898 | 0 | 168 | 7898 | 0.02 | 0.98 | 0.021 | 0.02 |
| 1 | 7730 | 0 | 359 | 7730 | 0.05 | 0.93 | 0.045 | 0.05 |
| 2 | 7371 | 558 | 103 | 7092 | 0.01 | 0.92 | 0.014 | 0.01 |
| 3 | 6710 | 2027 | 12 | 5696.5 | 0.00 | 0.92 | 0.002 | 0.00 |
| 4 | 4671 | 1519 | 5 | 3911.5 | 0.00 | 0.92 | 0.001 | 0.00 |
| 5 | 3147 | 2194 | 1 | 2050 | 0.00 | 0.92 | 0.000 | 0.00 |
| 6 | 952 | 952 | 0 | 476 | 0.00 | 0.92 | 0.000 | 0.00 |
| Obstructive bronchitis, 1^st^ diagnosis | | | | | | | | |
| Age | N at beginning of interval | N Censored | N events | N at risk ^a)^ | Proportion Terminating | CumulativeSurvival | Incidence  (Prob. Density) | Hazard Rate |
| 0 | 7898 | 0 | 105 | 7898 | 0.01 | 0.99 | 0.013 | 0.01 |
| 1 | 7793 | 0 | 388 | 7793 | 0.05 | 0.94 | 0.049 | 0.05 |
| 2 | 7405 | 544 | 306 | 7133 | 0.04 | 0.90 | 0.040 | 0.04 |
| 3 | 6555 | 1943 | 95 | 5583.5 | 0.02 | 0.88 | 0.015 | 0.02 |
| 4 | 4517 | 1471 | 50 | 3781.5 | 0.01 | 0.87 | 0.012 | 0.01 |
| 5 | 2996 | 2077 | 16 | 1957.5 | 0.01 | 0.86 | 0.007 | 0.01 |
| 6 | 903 | 903 | 0 | 451.5 | 0.00 | 0.86 | 0.000 | 0.00 |
| Asthma, 1^st^ diagnosis | | | | | | | | |
| Age | N at beginning of interval | N Censored | N events | N at risk ^a)^ | Proportion Terminating | CumulativeSurvival | Incidence  (Prob. Density) | Hazard rate |
| 0 | 7898 | 0 | 81 | 7898 | 0.01 | 0.99 | 0.010 | 0.01 |
| 1 | 7817 | 0 | 390 | 7817 | 0.05 | 0.94 | 0.049 | 0.05 |
| 2 | 7427 | 554 | 283 | 7150 | 0.04 | 0.90 | 0.037 | 0.04 |
| 3 | 6590 | 1967 | 97 | 5606.5 | 0.02 | 0.89 | 0.016 | 0.02 |
| 4 | 4526 | 1468 | 35 | 3792 | 0.01 | 0.88 | 0.008 | 0.01 |
| 5 | 3023 | 2083 | 11 | 1981.5 | 0.01 | 0.87 | 0.005 | 0.01 |
| 6 | 929 | 929 | 0 | 464.5 | 0.00 | 0.87 | 0.000 | 0.00 |
| 1^st^ move from birth-address | | | | | | | | |
| Age | N at beginning of interval | N Censored | N moved | N at risk ^a)^ | Proportion Terminating | CumulativeSurvival | Incidence  (Prob. Density) | Hazard rate |
| 0 | 7898 | 0 | 0 | 7898 | 0.00 | 1 | 0.000 | 0.00 |
| 1 | 7898 | 0 | 1538 | 7898 | 0.19 | 0.81 | 0.195 | 0.22 |
| 2 | 6360 | 0 | 1057 | 6360 | 0.17 | 0.67 | 0.134 | 0.18 |
| 3 | 5303 | 0 | 479 | 5303 | 0.09 | 0.61 | 0.061 | 0.09 |
| 4 | 4824 | 0 | 222 | 4824 | 0.05 | 0.58 | 0.028 | 0.05 |
| 5 | 4602 | 0 | 48 | 4602 | 0.01 | 0.58 | 0.006 | 0.01 |
| 6 | 4554 | 4554 | 0 | 2277 | 0.00 | 0.58 | 0.000 | 0.00 |

1. N at risk = N at beginning of age interval – ((n censured)/2)
