# Supplementary figures and images for: Asthma incidence in children growing up close to traffic: a registry-based birth cohort
Source: Environ Health. 2013 Oct 26;12:91. doi: 10.1186/1476-069X-12-91 (PMC4016196; doi:10.1186/1476-069X-12-91)

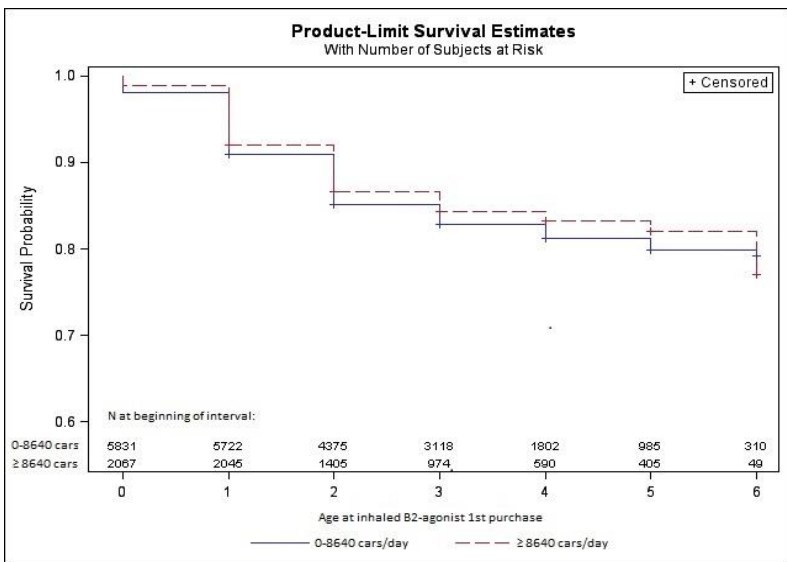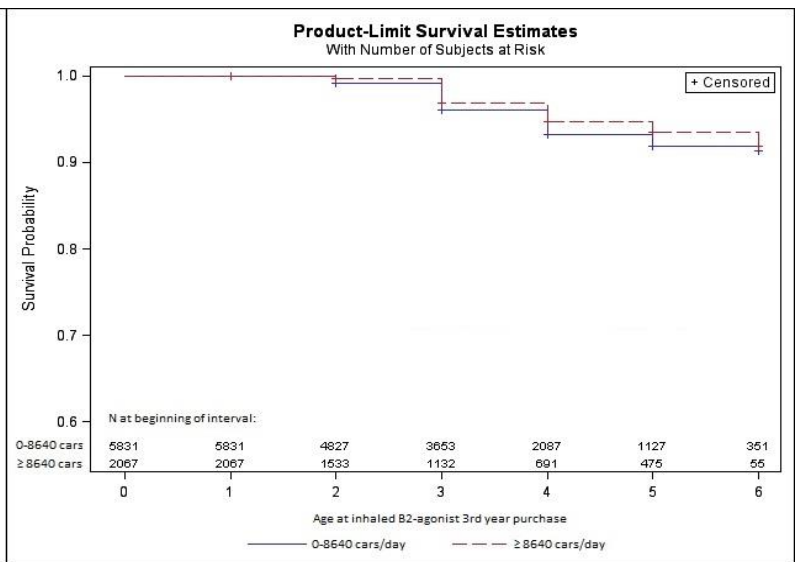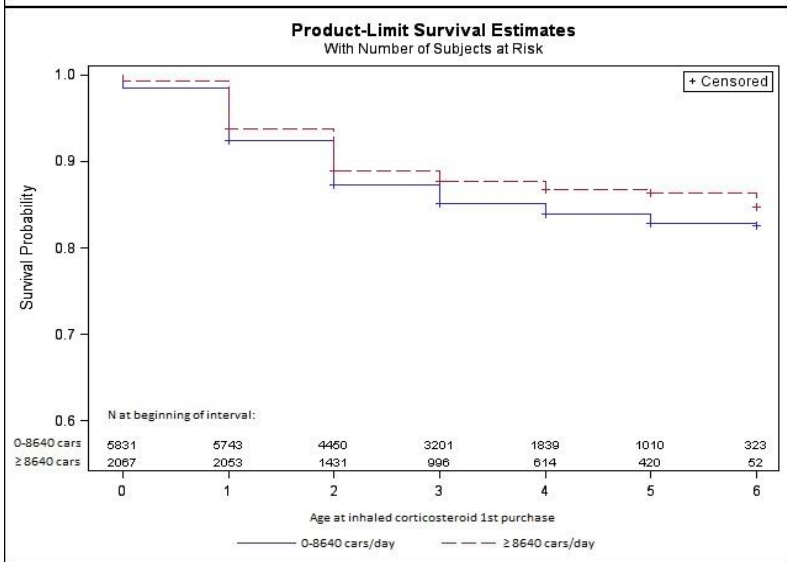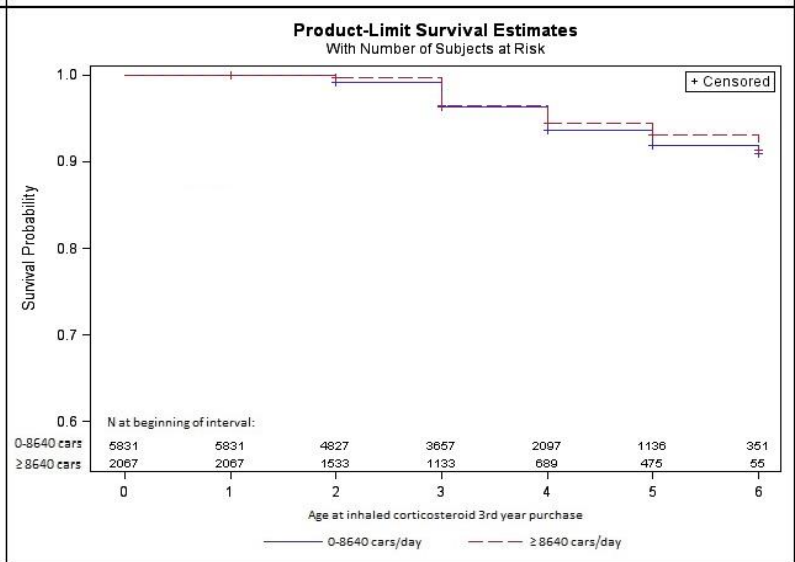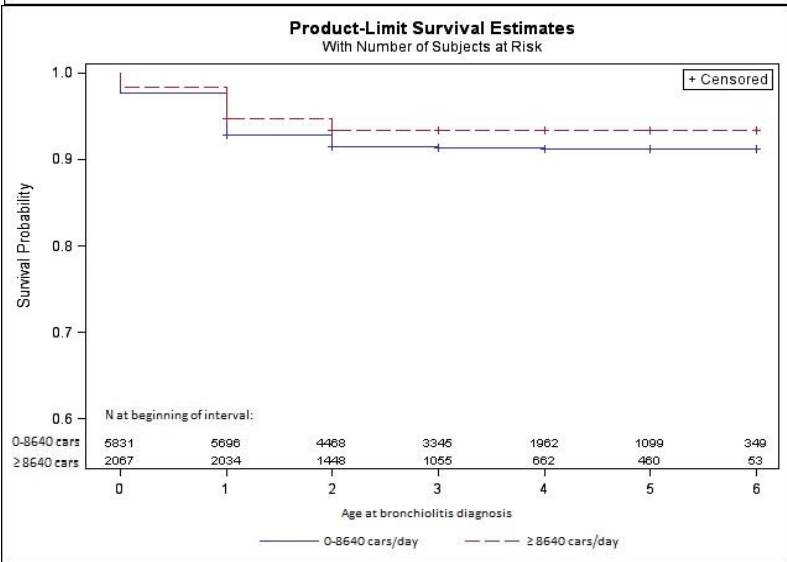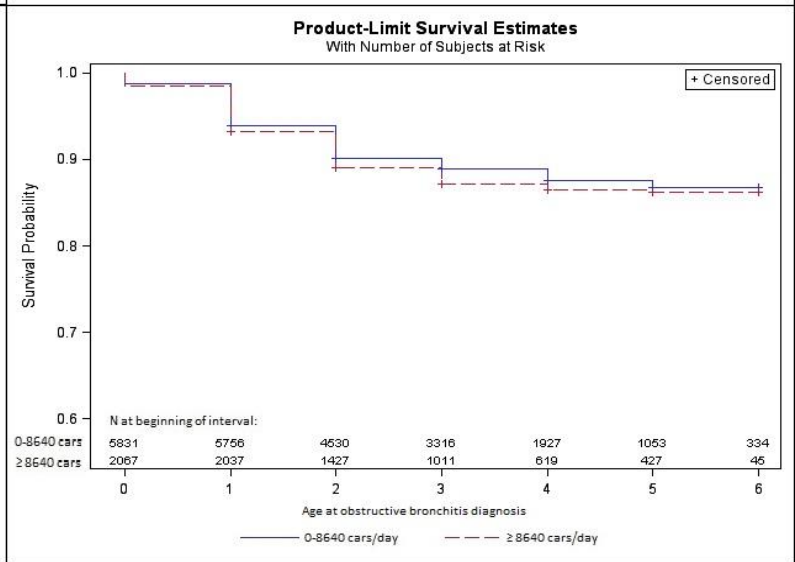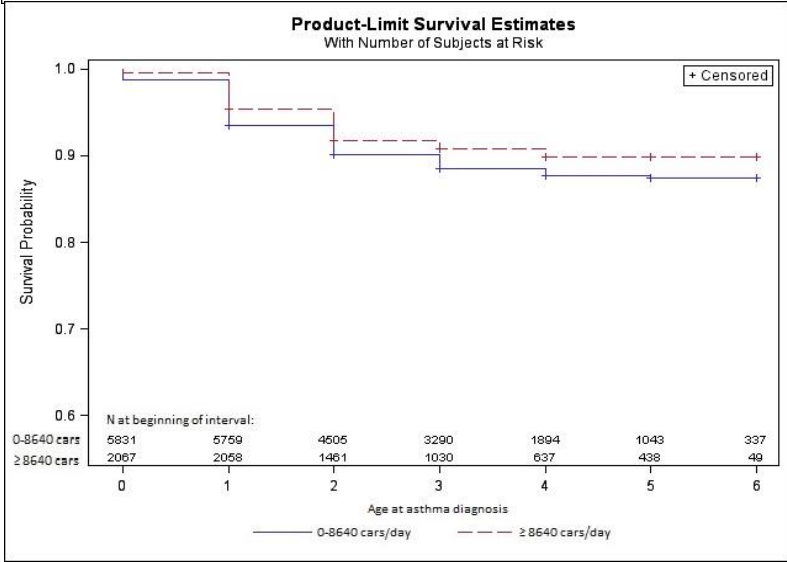

Additional file 2:  
Kaplan-Meier survival curves,  
for children who never moved  
during time at risk.

Supplement: Additional file 2 — Kaplan-Meier survival curves of asthma medication and diagnoses in relation to traffic intensity, with number at risk. [file 1476-069X-12-91-S2.pdf]
